# Biomarker MicroRNAs for Diagnosis, Prognosis and Treatment of Hepatocellular Carcinoma: A Functional Survey and Comparison

**DOI:** 10.1038/srep38311

**Published:** 2016-12-05

**Authors:** Sijia Shen, Yuxin Lin, Xuye Yuan, Li Shen, Jiajia Chen, Luonan Chen, Lei Qin, Bairong Shen

**Affiliations:** 1 Department of General Surgery, The First Affiliated Hospital of Soochow University, Suzhou 215006, China; 2 Center for Systems Biology, Soochow University, Suzhou, 215006, China; 3 Institute of Biological Sciences and Biotechnology, Donghua University, Shanghai, 201620, China; 4 Key Laboratory of Systems Biology, Chinese Academy of Sciences, Shanghai, 200031, China

## Abstract

Hepatocellular Carcinoma (HCC) is one of the most common malignant tumors with high incidence and mortality rate. Precision and effective biomarkers are therefore urgently needed for the early diagnosis and prognostic estimation. MicroRNAs (miRNAs) are important regulators which play functions in various cellular processes and biological activities. Accumulating evidence indicated that the abnormal expression of miRNAs are closely associated with HCC initiation and progression. Recently, many biomarker miRNAs for HCC have been identified from blood or tissues samples, however, the universality and specificity on clinicopathological features of them are less investigated. In this review, we comprehensively surveyed and compared the diagnostic, prognostic, and therapeutic roles of HCC biomarker miRNAs in blood and tissues based on the cancer hallmarks, etiological factors as well as ethnic groups, which will be helpful to the understanding of the pathogenesis of biomarker miRNAs in HCC development and further provide accurate clinical decisions for HCC diagnosis and treatment.

Hepatocellular Carcinoma (HCC) is the sixth most common cancer worldwide in terms of number of cases and the second major contributor to cancer mortality in man. The survival rates in the United States and developed countries are only 3% to 5%^1^,^2^. There are still no effective biomarkers for the early diagnosis and prognosis of HCC. Currently, only about 30% to 40% patients with HCC can get effective treatment at the right time^3^. It is extremely necessary to discover new biomarkers for precision diagnosis, prognosis and treatment of HCC.

MicroRNAs (miRNAs) are small endogenous non-coding RNAs with 22–24 nucleotides in length. They play important roles in regulating human genes by inhibiting translation or cleavage. Recent studies showed that miRNAs were associated with a variety of important biological processes such as cell proliferation, development, and apoptosis^4^,^5^. Accumulating evidence indicated that miRNAs could be latent biomarkers in human cancers, including gastric cancer, lung cancer, prostate cancer, and breast cancer etc.^6^,^7^,^8^,^9^. Nowadays, extensive research efforts have demonstrated the biomarker role of miRNAs in HCC. For example, Jiang and his colleagues confirmed that miRNA panel assay (miR-10b, miR-106b and miR-181a) could be potential biomarkers for HCC preliminary screening^10^. He *et al*. focused on the applications of miRNAs from 13 studies and 21 sets of data and the association between the risk of HCC and miRNAs polymorphisms^11^. Another review summarized the function of circulating miRNAs^12^, and a meta-analysis included 14 studies involving 1,848 cases with HCC and 1187 controls concluded that the miRNA panels can be biomarkers for HCC with AUC = 0.99 (96% sensitivity and 96% specificity)^13^. Many comprehensive reviews recommend to pay attentions to the role and function of miRNAs in disease diagnosis, prognosis and therapy^14^,^15^,^16^,^17^,^18^,^19^,^20^,^21^,^22^. However, the differences in biological features of miRNAs between blood and tissues are still unclear, which limits the investigation on understanding clinical implications of miRNAs in different specimen.

In this review, we performed comprehensive functional analyses and comparisons of miRNA biomarkers in blood and tissues. The miRNA biomarkers in “tissues” were mainly extracted from liver tissues, adjacent noncancerous tissues or human HCC tissues whereas those in “blood” were collected from plasma, serum or whole blood samples. This review aims at comprehensively understanding the pathogenic mechanism and clinical value of HCC biomarker miRNAs, and providing insights into precision diagnosis and treatment of HCC.

## Methods

### Data collection

We systematically collected HCC biomarker miRNAs from citations in NCBI PubMed by retrieval formula “(liver cancer[tiab] OR intrahepatic bile duct[tiab] OR hepatocellular carcinoma[tiab] OR hepatoblastoma[tiab] OR cholangiocarcinoma[tiab]) AND (miRNA* OR microRNA*) AND (biomarker*[tiab] OR marker*[tiab] OR indicator*[tiab] OR predictor*[tiab])”. Here, studies in which miRNAs were exactly defined as markers or biomarkers were mainly considered, and those identified from body fluids such as saliva, urine and sweat were excluded as we only focused on miRNA biomarkers in blood and tissues. Besides, for further comparing the differentiation between HCC and cirrhosis and providing valuable strategies for the early detection of HCC, we also collected diagnostic miRNA biomarkers for liver cirrhosis using retrieval formula “cirrhosis[tiab] AND diagnos*[tiab] AND (miRNA* OR microRNA*) AND (biomarker*[tiab] OR marker*[tiab] OR indicator*[tiab] OR predictor*[tiab])”.

### Target genes of miRNA biomarkers

The miRNA targets used in this study were integrated from both experimentally validated, *i.e.* miR2Disease^23^, TarBase (version 6.0)^24^, miRTarBase (version 4.5)^25^, miRecords (version 4.0)^26^ and computationally predicted, *i.e.* HOCTAR (version 2.0)^27^, ExprTargetDB^28^, and starBase (version 2.0)^29^ miRNA-target databases. To reduce false positives, we mainly selected miRNA-mRNA pairs validated by low-throughput experiments, *i.e.* real-time quantitative PCR, Western blot, etc. For computationally predicted pairs, they should reside in no fewer than two of the three prediction databases. Meanwhile, we unitized miRNA IDs according to the latest nomenclature in miRBase (release 21)^30^.

### Functional survey of HCC biomarker miRNAs

The functions of HCC biomarker miRNAs are summarized based on the hallmarks of cancers^31^,^32^. Since some of the miRNAs are associated with liver injury and few of the miRNAs’ functions are unclear, we therefore grouped their functions into 12 categories as antigrowth signals, resisting cell death, avoiding immune destruction, tissue invasion and metastasis, tumor promotion inflammation, sustained angiogenesis, limitless replicative potential, genome instability and mutation, other clinicopathological features, liver injury, tumor suppressor/onco-miR, and unclear. Moreover, we compared the pathogenesis of HCC biomarker miRNAs based on etiological factors as well as ethnic groups, *i.e.* the effects of Hepatitis B Virus (HBV), Hepatitis C Virus (HCV) and ethnic variation on HCC development.

### Pathway enrichment analyses

For better understanding the association between miRNAs and HCC pathogenesis, we mapped the targets of biomarker miRNAs onto signaling pathways using IPA (Ingenuity Pathway Analysis) program. The top 10 significantly enriched pathways (p-value < 0.01) were selected and further validated the correlation with HCC by PubMed literature exploration.

## Results

### Overview of the collected HCC biomarker miRNAs

After manually searching and checking in PubMed citations, a total of 50 and 18 diagnostic miRNA biomarkers in blood and tissues, respectively, were extracted from 44 articles (see Tables 1 and 2) and their clinicopathological features of HCC were further compared based on the hallmarks of cancer^31^, etiological factors and ethnic groups, respectively. As for prognostic and therapeutic biomarkers, respectively, 16 and 32 prognostic miRNAs in blood and tissues together with 8 therapeutic markers were collected according to records in 54 articles (see Tables 3, 4 and 5) and their clinicopathological features as well as functions were then explored.

### Functional characterization of HCC biomarker miRNAs based on cancer hallmarks

**Figure 1:**
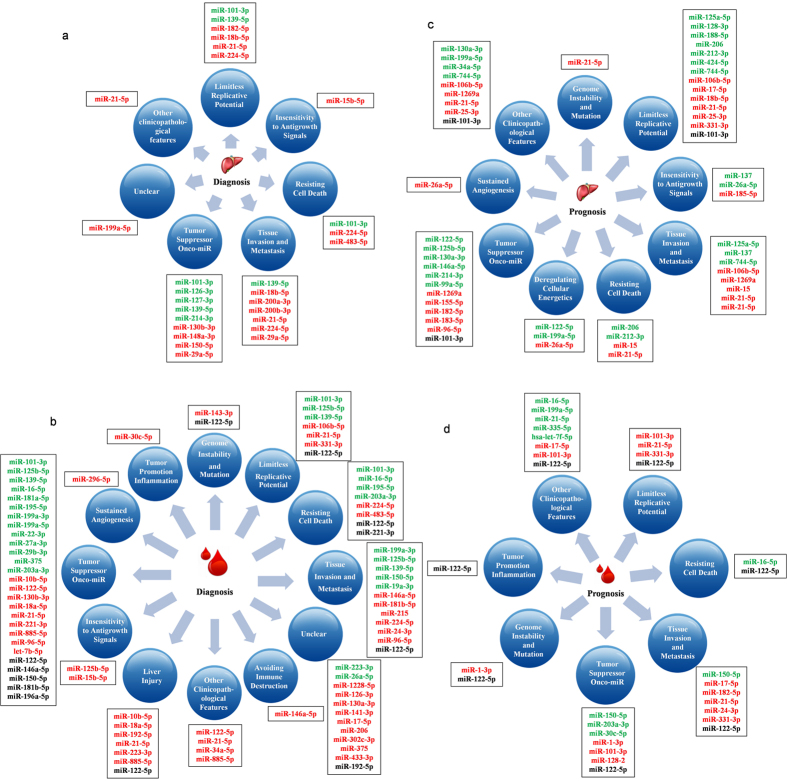
The correlation among clinicopathological features and reported HCC miRNA biomarkers. Here, miRNAs in red and green, respectively, represent the up and down-regulated expression in tissues and blood. The miRNA in black means that its expression can be inconsistently up- or down- regulated in different reports. Sub-figure (**a**,**b**) represent clinicopathological features of diagnostic miRNA biomarkers in tissues and blood, respectively. Sub-figure (**c**,**d**) represent clinicopathological features of prognostic miRNA biomarkers in tissues and blood, respectively.

The functional characterization of HCC biomarker miRNAs are summarized from the primary references and classified into 12 categories as shown in Fig. 1. It indicates that the biomarker miRNAs are associated with all aspects of hallmarks of cancers and all the hallmarks lead to the cancer. Therefore, the personalized biomarkers are needed to precision diagnosis, prognosis and treatment of the complex HCC. The functions of the biomarker miRNAs are summarized as follows.

### Insensitivity to Antigrowth Signals

Although it is unclear for the units and interconnections between the different kinds of antigrowth and differentiation-including signals and the core cell cycle machinery, an antigrowth signaling must be exist to circumvent developing HCC^31^. MiR-125b-5p and miR-15b-5p were the circulating diagnostic miRNA biomarkers associated with insensitivity to antigrowth signals and all of them were up-regulated and highly expressed in early-stage HCC cases^33^. Liu *et al*. combined miR-15b-5p and miR-130b-3p as a classifier for HCC detection, yielding a receiver operating characteristic curve area of 0.98 in their validation study, the same was found in tissue samples, miR-15-5p was also reported highly expressed^34^. As for prognostic biomarkers, three miRNAs related to insensitivity to antigrowth signals in the tissue samples were identified, including miR-137, miR-185-5p and miR-26a-5p. All of them were down-regulated in poor prognostic group which had a lower survival rate and shorter time to recurrence^35^,^36^,^37^.

### Resisting Cell Death

Cancer cells evolve various ways to circumvent or restrict apoptosis. The diversity of apoptosis-avoiding machinery and program reflects the multiplicity of apoptosis-including signals that tumor cell populations experienced while their evolution to the malignant state^32^. In tissues, miR-101-3p, miR-224-5p and miR-483-5p were associated with resisting cell death. Among them, miR-101-3p was down-regulated whereas the remaining two were reported to be up-regulated^38^,^39^,^40^. Resisting cell death was significantly associated with lower expression of miR-101-3p, miR-16-5p, miR-195-5p, miR-203a-3p and miR-221-3p in blood samples^38^,^41^,^42^,^43^. Increased miR-221-3p, miR-224-5p, miR-483-5p and miR-122-5p expression were also detected in blood of HCC patients^40^,^44^. These above diagnostic biomarkers as classifiers for HCC detection, yielding a receiver operating characteristic curve area of 0.635 to 0.884 (see Tables 1 and 2). On the other hand, miR-155-5p, miR-206, miR-21-5p and miR-212-3p could be recognized as biomarkers for HCC prognosis in tissues. The expression levels of miR-155-5p and miR-21-5p were up-regulated whereas others were down-regulated^45^,^46^,^47^,^48^. Circulating miR-122-5p and miR-16-5p could be used as putative biomarkers for HCC. Among them, miR-122-5p and miR-16-5p were shown to be up and down-regulated, respectively^49^,^50^.

### Avoiding Immune Destruction

According to the long-standing theory of immune surveillance proposes, most of solid tumors such as HCC appeared to have somehow controlled to avoid detection by the different kinds of arms of the immune system or could limit the extent of immunological killing, thus they could evade eradication by immune system^32^. Motawi and his colleagues overviewed that serum miR-146p-5p was up-regulated in HCC and showed the clinical value for HCV-related HCC diagnosis. This circulatory biomarker miRNA was reported to exerted negative effects on anti-tumor immune response^42^.

### Tissue Invasion and Metastasis

Invasion and metastasis, complex and multi-step processes, are elementary factors that affects HCC patients survival rate and their genetic and biochemical mechanisms remain poorly understood^31^. In tissues, high expression of miR-18b-5p, miR-200a-3p, miR-200b-3p, miR-21-5p, miR-224-5p and miR-29-5p were most frequently to be detected in HCC, and miR-139-5p was down-regulated. Therefore, they were valuable for diagnosis of HCC^39^,^51^,^52^,^53^,^54^,^55^,^56^,^57^. Several circulating miRNA biomarkers also displayed signally correlation with tissue invasion and metastasis, including highly expressed miR-146a-5p, miR-181b-5p, miR-182-5p, miR-21-5p, miR-215, miR-24-3p, miR-224-5p, miR-296-5p, miR-331-3p and miR-96-5p and low expressed miR-125b-5p, miR-199a-3p, miR-122-5p, miR-139-5p, miR-150-5p, miR-195-5p and miR-19a-3p. The above diagnostic biomarkers could be used as classifiers for HCC detection, yielding a receiver operating characteristic curve area of 0.645 to 0.943^42^,^51^,^55^,^56^,^58^,^59^,^60^,^61^,^62^,^63^.

In tissues, with regard to up-regulated microRNAs in HCC tissues, highly expression of miR-106b-5p, miR-155-5p, miR-17-5p, miR-182-5p, miR-183-5p, miR-18b-5p, miR-21-5p, miR-25-3p, miR-331-3p, miR-9-5p and miR-96-5p were significantly correlated with invasion and metastasis^45^,^47^,^52^,^56^,^64^,^65^,^66^,^67^,^68^,^69^,^70^,^71^. The expression level of miR-1269a in HCC patients without portal vein tumor embolus was reduced^72^. In addition, the low expression of miR-125a-5p, miR-128-3p, miR-137, miR-185-5p, miR-188-5p, miR-26a-5p, miR-503-5p and miR-744-5p were detected in HCC tissues compared with their non-tumor livers and were involved in the multi-step processes^35^,^36^,^37^,^73^,^74^,^75^,^76^. There were six circulating prognostic biomarker miRNAs reported to be associated with tissue invasion and metastasis, including miR-122-5p, miR-17-5p, miR-182-5p, miR-21-5p, miR-24-3p and miR-331-3p, all of them were up-regulated in the group with low survival rate^56^,^61^,^63^,^77^,^78^. Meanwhile, the serum miR-150-5p was shown highly expressed in HCC patients after surgical operation and then low expressed after tumor relapsed^60^.

### Tumor Promoted Inflammation

Inflammation has been proved to be existed at the earliest stage of tumor processes and to be capable of fostering the progression of incipient neoplasia into advanced tumors^79^. Besides chemicals, particularly reactive oxygen species were positively mutagenic for adjacent cancer cells, accelerating their genetic evolution towards the high malignant carcinoma^80^. In blood, the increased expression of miR-30c-5p could be used as a new classifier for HCV-positive HCC in early-stage^81^. In addition, hepatic necroinflammatory activity was associated with the high expression of miR-122-5p in plasma. The over expression of circulating miR-122-5p was a prognostic biomarker predicting the poor survival rate of patients underwent radio frequency ablation^49^.

### Sustained Angiogenesis

Both oxygen and nutrients transported by vasculature are essential for cell survival and function. All cells in tissues obligate to live within 100 μm of a capillary blood vessel. The evidence showed that cells with aberrant proliferative lesions tended to lack angiogenic ability at first, and led to hinder the capability for expansion^31^. The development of angiogenic ability is vital for incipient neoplasia growth^82^,^83^. The over expression of circulating miR-296-5p was significantly associated with tumor angiogenesis^42^. In tissues, high expression of miR-26a-5p could suppress tumor angiogenesis in HCC by targeting HGF-cMet signaling, and it was a novel prognostic biomarker for HCC^84^.

### Limitless Replicative Potential

There are three factors can lead to an uncoupling of the growth of a cell process from signals in their microenvironment, including insensitivity to antigrowth signals, resistance to apoptosis, and growth signal autonomy. Senescence, just like apoptosis, is as a protective system that could be activated by opposite growth signals or shortened telomeres that drives abnormal cells irreversibly into a G0-like state, and it could prevent further proliferation^31^. High expression of miR-182-5p, miR-18b-5p, miR-21-5p and miR-224-5p, together with the down-regulated expression of miR-101-3p and miR-139-5p not only played important roles in the regulation of cell proliferation and limitless replicative potential, but also were diagnostic signals for HCC^38^,^39^,^51^,^52^,^54^,^55^,^56^,^85^. High expression of miR-106b-5p, miR-21-5p, miR-331-3p and low expression of miR-101-3p, miR-125b-5p, miR-139-5p had great potential to be noninvasive and accurate circulating biomarkers for HCC preliminary screening^10^,^38^,^51^,^55^,^56^,^61^. Moreover, some opposite results about the expression levels of miR-122-5p were discussed^44^,^86^. In tissues, high expression of eight miRNAs (*i.e.* miR-101-3p, miR-106-5p, miR-17-5p, miR-18b-5p, miR-21-5p, miR-25-3p and miR-331-3p) and low expression of seven miRNAs (*i.e.* miR-125a-5p, miR-128-3p, miR-188-5p, miR-206, miR-212-3p, miR-424-5p and miR-744-5p) were outstandingly correlated with limitless replicative potential and could provide positive prognostic values for HCC^38^,^46^,^47^,^48^,^52^,^56^,^64^,^65^,^69^,^70^,^73^,^74^,^76^,^87^. Four prognostic circulating miRNAs associated with proliferation and limitless replicative potential, including miR-101-3p, miR-122-5p, miR-21-5p and miR-331-3p, were reported up-regulated in HCC patients^38^,^56^,^61^,^77^.

### Genome Instability and Mutation

Multi-step cancer progression could be described as a series of genic clonal expansions. Acquiring the chance of an enabling mutant gene triggered these clonal expansions^88^,^89^,^90^. The widespread destabilization of genome is inherent to the vast majority of HCC cells^32^. The high expression of miR-122-5p and low expression of miR-143-3p in blood were prominently correlated with differentiation and genome instability. They could be used as noninvasive circulating biomarkers for diagnosis of HCC^59^,^62^,^86^. Up-regulated expression of miR-21-5p has been observed to be associated with genome instability and mutation, and it was a novel prognostic biomarker for HCC^68^. Patients with high serum concentrations of miR-1-3p and miR-122-5p showed a long overall survival time and these miRNAs could be used to assess the HCC staging scores^77^,^91^.

### Liver injury

Biochemical molecules including miRNAs can be released into the circulation system due to the hypoxia and damage of liver cells. Accumulating reports indicated that serum miR-10b-5p, miR-122-5p, miR-18-5p, miR-192-5p, miR-21-5p, miR-223-3p and miR-885-5p were went up in patients with chronic hepatitis or HCC and they could serve as diagnostic biomarkers for liver injury but not specific for HCC^10^,^42^,^92^,^93^,^94^.

### Tumor suppressor/onco-miR

Genetic suppressor and carcinogenicity interpreted the function of miRNAs from another perspective. In tissues, high expression of miR-150-5p and miR-29a-5p and low expression of miR-101-3p, miR-126-3p, miR-127-3p, miR-139-5p and miR-214-3p played tumor-suppressor roles and could be used as diagnostic biomarkers for HCC^38^,^39^,^51^,^57^,^95^,^96^,^97^. The circulating miR-101-3p, miR-122-5p, miR-125b-5p, miR-139-5p, miR-150-5p, miR-16-5p, miR-181a-5p, miR-199a-3p, miR-199a-5p, miR-203a-3p, miR-21-5p, miR-22-3p, miR-29b-3p, miR-375, let-7b-5p correlated with tumor suppressor and could be potential biomarkers to differentiate HCC from healthy controls^10^,^38^,^41^,^44^,^51^,^58^,^59^,^60^,^86^,^97^,^98^. On the other hand, miR-101-3p, miR-122-5p, miR-125b-5p, miR-130a-3p, miR-146a-5p, miR-214-3p and miR-99a-5p were considered as tumor suppressors in HCC and served as prognostic indicators for HCC^38^,^99^,^100^,^101^,^102^,^103^,^104^. Serum miR-1-3p, miR-101-3p, miR-122-5p, miR-150-5p, miR-203a-3p and miR-30c-5p were associated with suppressing tumorigenicity and new independent parameters of overall survival in HCC^38^,^49^,^60^,^77^,^91^,^105^.

The high expression of miR-130b-3p, miR-148a-3p, miR-181b-5p, miR-221-3p, miR-885-5p and miR-96-5p were functional in tumorigenicity and could be served as early diagnostic biomarkers for different tumor type^34^,^106^. Meanwhile, miR-10b-5p, miR-130b-3p, miR-146a-5p, miR-18-5p, miR-195-5p, miR-196a-5p and miR-27a-3p were related to carcinogenicity and played vital roles in HCC detection^10^,^34^,^42^,^59^,^94^,^107^. There were six miRNAs associated with oncogenicity and could be potential biomarkers for the overall survival of patients with HCC, including miR-1269a, miR-155-5p, miR-182-5p, miR-183-5p, miR-96-5p and miR-128-2^66^,^72^,^108^,^109^.

### Other clinicopathological features

Besides the above ten clinicopathological features and the hallmarks of cancer, biomarker miRNAs were also correlated with other clinicopathological features, such as secretion by primary cancer cells, child stage, cholesterol reverse transport, tumor size and recurrence, etc. Tomimaru *et al*. found that miR-21-5p was excessively secreted by primary cancer cells and could be a potential diagnostic biomarker for HCC^110^. Motawi and his colleagues identified that serum miR-34a-5p was correlated with child stage and BCLC score and could be used as an early biomarkers for HCC in high-risk group^42^. The miR-885-5p and miR-122-5p in serum was reported related to cholesterol reverse transport and assessment of liver pathologies^111^. In addition, miR-101-3p, miR-106b-5p, miR-130a-3p, miR-16-5p, miR-199a-5p, let-7f-5p and miR-34a-5p were found to have a significant correlation with tumor size in the tissue and serum of HCC patients^50^,^64^,^102^,^112^,^113^,^114^. The present literature also provided evidence that miR-130a-3p, miR-21-5p, miR-25-3p, miR-17-5p were independent prognostic factors and were associated with the TNM classification which is a universally accepted cancer staging system based on extension and size of the primary tumor (T), the adjacent lymph node (N), and the distant metastasis (M)^68^,^69^,^78^,^102^. The down-regulated expression of miR-774-5p and let-7f-5p can be considered as noninvasive biomarkers for predicting of the recurrence of HCC^76^,^114^.

### Comparison of HCC biomarker miRNAs based on etiological factors and ethnic groups

Recently, accumulating evidence indicated that the occurrence and development of HCC are closely associated with etiological factors as well as ethnic groups. The differentiation between HCC and liver cirrhosis, for instance, is one of the main problems for the early detection of HCC. Moreover, different etiological factors such as HBV (Hepatitis B Virus) and HCV (Hepatitis C Virus) can also contribute to the HCC carcinogenesis. On the other hand, the incidence and mortality of HCC often showed different patterns among different ethnic groups. Hence it is necessary to compare HCC biomarker miRNAs based on etiological factors and ethnic groups.

### Biomarker miRNAs for classifying of HCC and liver cirrhosis

**Figure 2:**
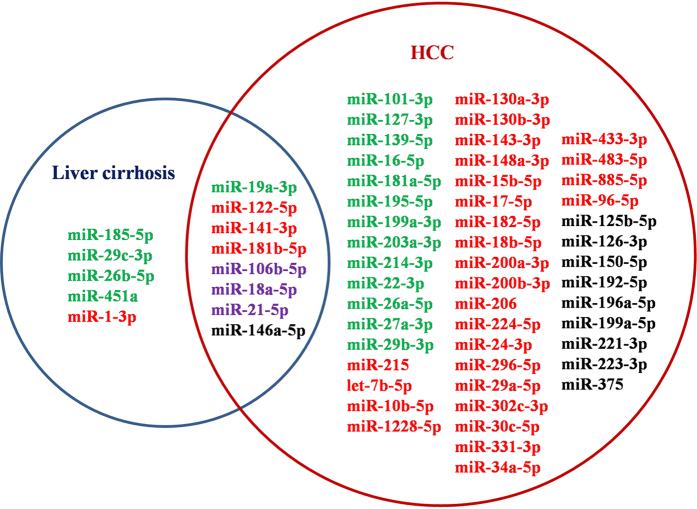
The Venn diagram of miRNA biomarkers for liver cirrhosis and HCC. Here circles in blue and red, respectively, represent miRNAs for cirrhosis and HCC. The miRNAs in red and green represent the up- and down-regulated expression, respectively. The miRNAs in purple means they showed inverse expression patterns in cirrhosis and HCC samples and those in black means their expressions were inconsistently up- or down- regulated according to different literature reports.

After manually searching for citations in PubMed, a total of 13 miRNA biomarkers for liver cirrhosis diagnosis were collected (see Table S1). We then compared them with HCC diagnostic miRNA biomarkers in order to screen key signatures for HCC early detection. As shown in Fig. 2, eight miRNAs, *i.e.* miR-106b-5p, miR-122-5p, miR-141-3p, miR-146a-5p, miR-181b-5p, miR-18a-5p, miR-19a-3p and miR-21-5p, were shared by cirrhosis and HCC. Interestingly, three of them (miR-106b-5p, miR-18a-5p and miR-21-5p) showed inverse expression patterns in cirrhosis and HCC groups. For example, the expression of miR-106b-5p (miR-106b) was down in cirrhosis samples^115^ whereas it turned out to be up-regulated in the blood of HCC patients^10^. In addition, miR-19a-3p (miR-19a) was reported as a useful molecular marker for monitoring the progression of liver fibrosis to cirrhosis and finally, to HCC^42^.

The remaining 5 and 49 miRNAs, respectively, were specific to cirrhosis and HCC, which could be served as independent factors for classifying of cirrhosis and HCC. For example, miR-29c-3p showed significant positive correlations with the level of serum cholinesterase (CHE) and albumin (ALB) in liver cirrhosis patients, suggesting that the miRNA played functional roles in the establishment of liver cirrhosis^116^. Han *et al*. found that two miRNAs, *i.e.* miR-224 (miR-224-5p) and miR-214 (miR-214-3p), were significantly up- and down-regulated in HCC tissue samples respectively, which provided novel biomarker signatures for HCC diagnosis and treatment^39^.

It can be concluded that biomarker miRNAs revealed the pathogenesis of cirrhosis and HCC at the post-transcriptional level and could help deeply understand the differentiation between cirrhosis and HCC. From the perspective of precision medicine, HCC miRNA biomarkers, especially those specific to HCC, were indicators for capturing the early diagnostic signatures at the time of HCC initiation.

### Biomarker miRNAs for monitoring the development of HBV/HCV-related HCC

**Figure 3:**
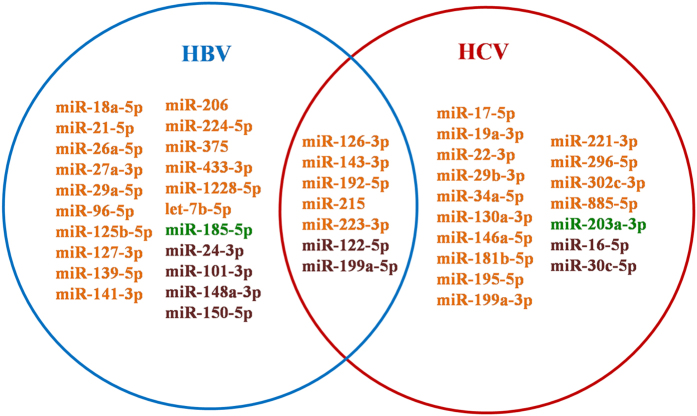
The Venn diagram of miRNA biomarkers for HBV/HCV-related HCC. Here miRNA biomarkers for HBV/HCV-related HCC were extracted from our collected dataset. Circles in blue and red, respectively, represent miRNAs for HBV-related HCC and HCV-related HCC. The miRNAs in orange and dark green represent the diagnostic and prognostic markers, respectively. The miRNAs in brown means they had both diagnostic and prognostic role according to different literature reports.

It has been widely acknowledged that the progression of HCC is closely affected by the infection of etiological factors, such as HBV, HCV, etc. On the other hand, miRNAs are reported to play crucial roles in HBV/HCV replication and pathogenesis^117^,^118^,^119^, *i.e.* they regulated HBV by directly binding to HBV transcripts or changing HBV gene expression at the transcriptional level^118^. For better investigating the influence of HBV/HCV on HCC development, miRNA biomarkers for HBV/HCV-related HCC were extracted from our collected dataset. As illustrated in Fig. 3, several miRNAs, *i.e.* miR-122-5p, miR-126-3p, miR-143-3p, miR-192-5p, etc., were functional in both HBV- and HCV-related HCC evolutionary progression. For example, Tan *et al*. found that serum miR-122-5p could be used as the diagnostic biomarker for detecting HBV-related HCC. Both the area under the receiver operating characteristic curve (AUC) and logistic regression model convinced the predictive power^86^. Meanwhile, the miRNA was also turned out to be effective for early detection of HCC on top HCV infection. Using the miRNA panel where miR-122-5p included, HCC patients could be classified from healthy controls and liver cirrhosis patients with high diagnostic accuracy^120^.

There is still a large number of biomarker miRNAs that could be specifically used for monitoring the development of HBV/HCV-related HCC. Chen *et al*. analyzed the plasma samples from 242 individuals and uncovered that the expression of miR-125b-5p (miR-125b) was significantly down-regulated in HBV-induced HCC (HBV-HCC) patients compared to healthy controls as well as HBV groups without HCC^121^. Moreover, the low plasma level of miR-125b-5p also reflected the higher possibility of metastasis. Therefore, the miRNA held promise as a valuable diagnostic biomarker for HBV-HCC and HBV-infected patients with high HCC risks could be early detected by dynamically monitoring the changes of this miRNA. Liu *et al*. demonstrated that the expression levels of miR-30c-5p (miR-30c) and miR-203a-3p (miR-203a) were crucial indicators for predicting the poor prognosis of HCV-related HCC because the core protein of HCV could down-regulate the expression of miR-30c-5p and miR-203a-3p, resulting in the activation of epithelial-mesenchymal transition in normal hepatocytes as well as HCC tumor cells. As reported before, the activation process may contribute to the carcinogenesis of HCC^105^.

Understanding the pathogenesis of miRNA biomarkers in HBV/HCV-related HCC provided insights to evaluate the potential effects of HBV/HCV on HCC development, which will be helpful to the early and personalized detection of HCC.

### HCC miRNA biomarkers within different ethnic groups

**Figure 4:**
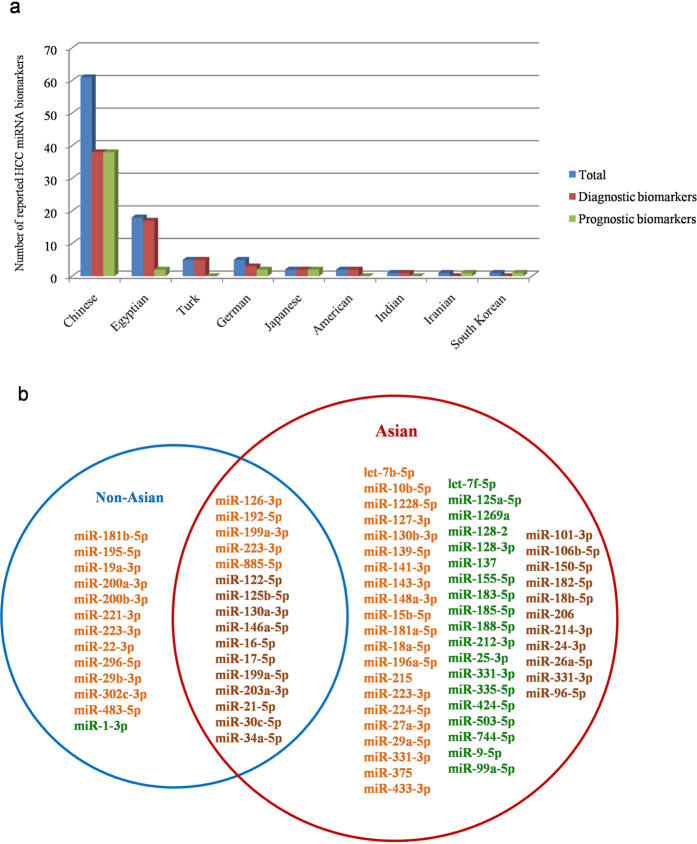
HCC miRNA biomarkers in different ethnic groups. Here miRNA biomarkers were classified based on the race/nation of patients described in each citation. Sub-figure (**a**) represents the distribution of reported HCC miRNA biomarkers in different national cohorts. Bars in blue, red and green mean the number of total, diagnostic and prognostic miRNA biomarkers, respectively. Sub-figure (**b**) is the Venn diagram of HCC miRNA biomarkers for Asian and non-Asian respectively. Circles in blue and red, respectively, represent Asian-related and non-Asian-related miRNA biomarkers. The miRNAs in orange and dark green represent the diagnostic and prognostic markers, respectively. The miRNAs in brown means they had both diagnostic and prognostic role according to different literature reports.

Genomic profiling of HCC tumors showed that HCC patients in different geographic regions tended to have specific recurrent molecular aberrations^122^. Asians, on the whole, achieved the highest HCC incidence according to the report by Wong *et al*.^123^. In terms of prognosis, the overall survival rate was also disparate among different ethnic groups^124^. Here we reorganized HCC miRNA biomarkers based on the ethnicity of patients described in each citation. As illustrated in Fig. 4a, most of the reported HCC miRNA biomarkers were related to Chinese population, which indirectly indicated the high risk or high incidence of HCC in China. For further exploring the ethnic specificity of HCC miRNA biomarkers, we then partitioned miRNAs into two categories based on the patient race, *i.e.* Asian-related (Chinese, Japanese, South Korean, Indian and Iranian) and non-Asian-related (Egyptian, American, Turk and German) HCC miRNA biomarkers. As shown in Fig. 4b, the number of Asian-specific HCC miRNA biomarkers is far more than that of non-Asian. We noticed that some miRNAs were reported to be functional in both Asian and non-Asian group. However, the expression pattern of them was sometimes quite different when they were involved in different pathogenic processes or belonged to different ethnic groups. For example, miR-125b-5p was associated with the biological behavior of HCC and had the diagnostic value of HCC for both Turks and Chinese. As in plasma samples of Chinese patients, it was found to be down-regulated^121^ whereas in Turks samples, its expression level was up^33^. For comparison of Egyptian and Chinese, the down-regulation of miR-146a-5p was correlated with HCC carcinogenesis and deterioration in Chinese population^103^, but in samples of Egyptian patients, it was inverse^42^.

This ethnic difference may be caused by the heterogeneous pathogenesis, lifestyles and various factors including the diet, environmental exposures, *etc.* Moreover, the incidence of HBV/HCV infection in different countries is also inconsistent. Therefore, more in-depth researches on ethnically specific miRNA biomarkers is of clinical significance, which would provide personalized strategies for HCC diagnosis and treatment in the era of precision medicine.

### Pathway enrichment analysis for targets of HCC miRNA biomarkers

**Figure 5:**
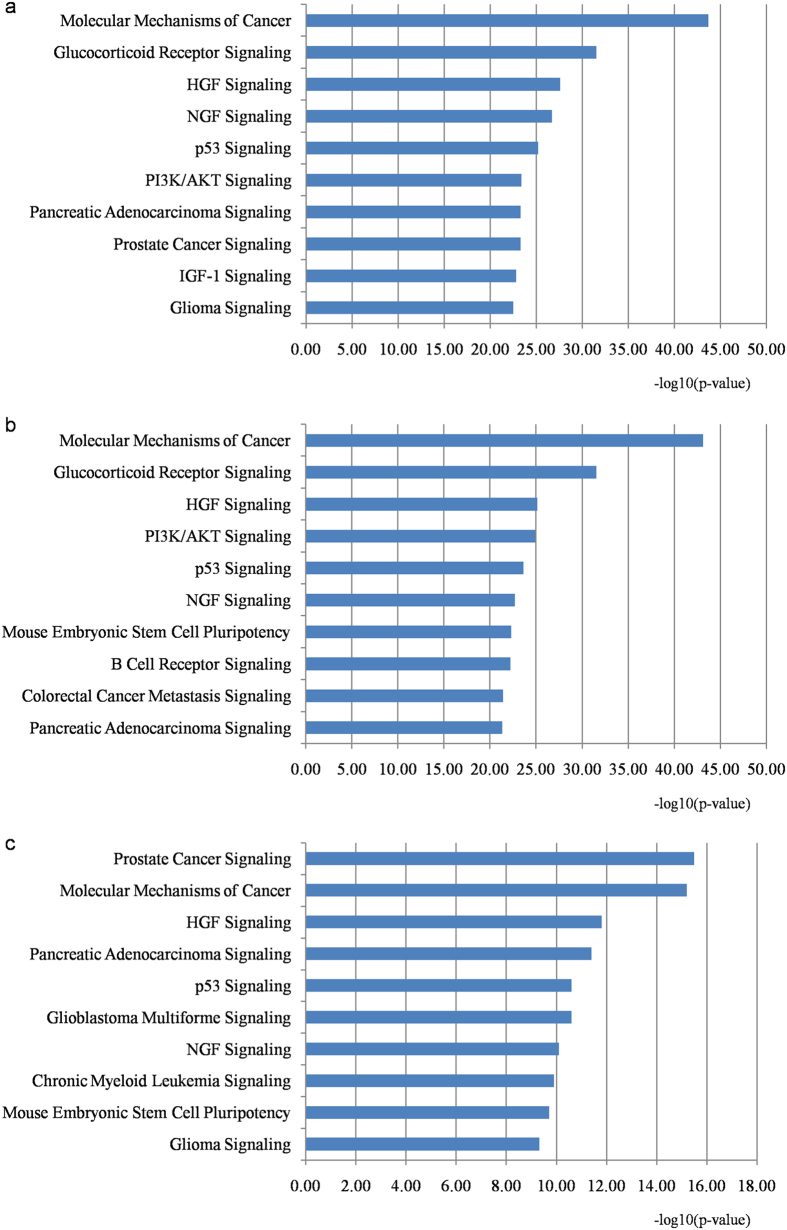
Top 10 pathways significantly enriched with targets of different biomarker miRNAs from HCC tissue and blood. Sub-figure (**a**), (**b**), and (**c**) represent pathways enriched by targets of diagnostic, prognostic and therapeutic biomarker miRNAs, respectively. The statistical significance level (p-value) was negative 10-based log transformed.

We performed the pathway enrichment analysis for targets of different types of reported miRNA biomarkers using IPA program. Here the targets of miRNA biomarkers originated from seven publicly available miRNA-target databases, including four experimentally validated databases and three computationally predicted databases (see Methods). For the three categories, *i.e.* the diagnostic, prognostic and therapeutic biomarker miRNAs, the top 10 significantly enriched pathways (p-value < 0.01) were chosen and shown in Fig. 5. The common enriched pathways among them were Molecular Mechanisms of Cancer, Glucocorticoid Receptor Signaling, HGF Signaling, NGF Signaling, p53 Signaling etc. Most of them are well-studied cancer associated pathways. Das *et al*. reported that the pathway Molecular Mechanisms of Cancer was potentially associated with recurrent HCC secondary to HCV following liver transplantation^125^. Glucocorticoids are involved in controlling many essential biological processes that are related to energy supply and growth control. The Glucocorticoid Receptor often functions as a cofactor of transcription factor STAT5 for growth hormone induced genes and Glucocorticoid Receptor Signaling has been turned out to be important in body growth, steatosis and metabolic liver cancer development^126^. The experimental result in mouse model demonstrated that the metabolic dysfunction and impairment of Glucocorticoid Receptor Signaling could cause steatosis and HCC in mice^127^. Wu *et al*. revealed that the HGF signaling could be activated by over expression of gene C1GALT1 in HCC via modulation of MET O-glycosylation and dimerization, which offered new insights into O-glycosylation and HCC pathogenesis^128^. Jin *et al*. indicated that p53 Signaling pathway was significantly dysregulated in HCC and it could reflect the development and progression of HCC^129^. Moreover, a number of genes participated in regulating human HCC by interacting with p53 Signaling pathway. For instance, the key gene RASSF10, which is located on chromosome 11p15.2, could suppress the growth of HCC via activating p53 Signaling pathway^130^. EGR1 is one of the key components in p53 Signaling, the re-expression of gene BCL6B in HCC cells could increase its expression and finally contribute to the activation of p53 Signaling^131^.

## Discussion

In this review, we made comprehensive functional survey and comparison of HCC diagnostic, prognostic and therapeutic miRNAs in blood and tissues. The number of diagnostic miRNA biomarkers in blood is approximately twice as much as those in tissues and meanwhile, the number of prognostic miRNA biomarkers in tissues is twice as much as those in blood. The reason for the statistical difference may be that many studies are inclined to investigate the noninvasive diagnostic miRNA biomarkers and researchers tend to use relatively stable hepatogenic biomarkers as prognostic indicators because miRNAs may be released into the blood selectively^132^,^133^. Most of the diagnostic, prognostic and therapeutic miRNA biomarkers are associated with one or two clinic pathological features in blood and tissues. A great number of prognostic biomarkers with high expression levels were detected in patients with shorter overall survival. Since the etiological factors as well as ethnic groups are closely associated with HCC carcinogenesis, we analyzed miRNA biomarkers by taking the HBV/HCV infection as well as regional variations into account in order to provide better clues for HCC pathogenic research. We mainly selected miRNAs which were explicitly reported as HCC markers/biomarkers in our current study. Besides, several miRNAs are still common and important during HCC development. For example, miR-142-3p was functional in HCC tumorigenesis and played a key role in regulating human RAC1 gene. The upregulation of miR-142-3p inhibited the expression level of RAC1 mRNA, suppressing the migration and invasion of HCC cells^134^. Interferon regulatory factor-1 (IRF-1) is a tumor-suppressor in HCC and its down-expression would help HCC tumors evade death. Yan *et al*. found that miR-23a was a negative regulator of IRF-1in HCC, which highlighted its importance in HCC initiation and progression^135^. Zhang *et al*. demonstrated that miR-99a could directly regulate AGO2 and control tumor growth in HCC, indicating the potential strategies for HCC treatment^136^.

HCC is a complex disease which is difficult for early diagnosis and treatment. The death rate of HCC remains high due to its poor prognosis. To some extent, miRNAs are effective biomarkers for HCC because of the noninvasive detection, good specificity and sensitivity. More systematic investigations and clinical experiments need to be done for better understanding the role and function of miRNA biomarkers in HCC pathogenesis^137^,^138^,^139^.

## Additional Information

**How to cite this article**: Shen, S. *et al*. Biomarker MicroRNAs for Diagnosis, Prognosis and Treatment of Hepatocellular Carcinoma: A Functional Survey and Comparison. *Sci. Rep.* **6**, 38311; doi: 10.1038/srep38311 (2016).

**Publisher's note:** Springer Nature remains neutral with regard to jurisdictional claims in published maps and institutional affiliations.

## Supplementary Material

Supplementary Table

## Figures and Tables

**Table 1:** Diagnostic biomarkers in tissues for hepatocellular carcinoma.

Reported ID	Offical ID	Sample	Ethnicity	Features	Expression	AUC	PMID	Validated Targets
miR-101	miR-101-3p	30 HC 67 CHB 61 HBV-LC 67 HBV-HCC	China	1.inhibit HCC cell proliferation2.tumor suppressor3.promote apoptosis	down	CHB from HC 0.635 HBV-LC from HC 0.884 HBV-HCC from HC 0.788	24971953^38^	Mcl-1, SOX9
miR-126	miR-126-3p	19 HCV 6 HCC	Germany	tumor suppressor	down	NA	25500075^95^	NA
miR-127	miR-127-3p	33 HCC	China	tumor suppressor	down	NA	24854842^96^	NA
miR-130b	miR-130b-3p	97 HCC	China	onco-miR	up	0.914	22403344^34^	RUNX3
miR-139	miR-139-5p	31 CHB 31 HCC	China	1.suppress metastasis and progression of cancer cells2.tumor suppressor	down	HCC from CH 0.761 (0.770^1^)	24549282^51^	Rho-kinase 2
miR-148a	miR-148a-3p	19 HCC	China	onco-miR	up	NA	22496917^106^	NA
miR-150	miR-150-5p	15 HC 15 ICC	China	tumor suppressor	up	0.764	25482320^97^	NA
miR-15b	miR-15b-5p	96 HCC	China	preventing replicative stress in response to mitogenicsignalling	up	0.98^2^	22403344^34^	NA
miR-182	miR-182-5p	HCC	China	proliferation	up	NA	24653623^85^	IGF1R and GSK3B
miR-18b	miR-18b-5p	110 HCC	Japan	1.proliferation2.loss of cell adhesion ability	up	NA	23496901^52^	TNRC6B
miR-199a	miR-199a-5p	17 CH 23 HCC	Egypt	NA	down	0.856	26302751^54^	Mitogen-activated protein kinase (MAPK)
miR-200a	miR-200a-3p	29 HCC	Germany	suppress cancer cell migration	up	NA	24895326^53^	ZEB1/ZEB2
miR-200b	miR-200b-3p	29 HCC	Germany	suppress cancer cell migration	up	NA	24895326^53^	ZEB1/ZEB2
miR-21	miR-21-5p	50 HC 30 LC 136 HCC	Japan	excessive secretion by primary cancer cells	up	CH from HC 0.773 HCC from HC 0.953	21749846^110^	NA
miR-21	miR-21-5p	17 CH 23 HCC	Egypt	1.cell growth2.migration3.invasion	up	0.943	26302751^54^	phosphatase and tensin homolog (PTEN)
miR-21	miR-21-5p	30 HC 97 HCC	China	1.promote cell proliferation2.tumor invasion	up	NA	25973032^55^	PDCD4 and PTEN
miR-21	miR-21-5p	74 ICC	China	intrahepatic cholangiocarcinoma proliferation and growth	up	NA	25803229^56^	PTPN14 and PTEN
miR-214	miR-214-3p	9 HC 10 HCC	China	tumor suppressor	down	NA	24789420^39^	EZH2, CTNNB1 and CDH1
miR-224	miR-224-5p	9 HC 10 HCC	China	1.cell proliferation2.migration3.invasion4.anti-apoptosis	up	NA	24789420^39^	CD40
miR-29a-5p	miR-29a-5p	266 HCC	China	1.tissue invasiveness and metastasis r2.tumor suppresso	up	0.746	23285022^57^	NA
miR-483-5p	miR-483-5p	69 HC 69 HCC	America	anti-apoptotic oncogene	up	HCC from HC 0.827	24127413^40^	NA

Abbreviations and note: HC: healthy controls; CHB: patients with chronic type B hepatitis; CH: chronic hepatitis; HCV: hepatitis C virus; HCC: hepatocellular carcinoma; LC: liver cirrhosis; HBV: hepatitis B virus; ICC: Intrahepatic cholangiocarcinoma; NA: not available; 1: combination of plasma miRNA-139 with serum AFP; 2: combined miR-15b and miR-130b.

**Table 2:** Diagnostic biomarkers in blood for hepatocellular carcinoma.

Reported ID	Offical ID	Sample	Source	Ethnicity	Features	Expression	AUC	PMID	Validated Targets
miR-199a-3p	miR-199a-3p	156 HC 78 HCC	serum	China	invasion capability	down	0.883	25618599^58^	phosphorylated-S6 protein
miR-223	miR-223-3p	167 HC 169 CHB 141 LC 457 HCC	blood	China	NA	down	0.864(training set) 0.888(validation set)	22105822^59^	Stathmin1
miR-101	miR-101-3p	30 HC 79 CHB 61 HBV-LC 67 HBV-HCC	serum	China	1.inhibit HCC cell proliferation2.tumor suppressor3.promote apoptosis	down^1^	CHB from HC 0.635 HBV-LC from HC 0.884 HBV-HCC from HC 0.788	24971953^38^	Mcl-1, SOX9
miR-106b	miR-106b-5p	50 HC 31 CLD 27 HCC	blood	China	Proliferation	up	HCC from HC 0.89 HCC from CLD 0.81 CLD from HC 0.63	25761179^10^	p21/E2F5
miR-10b	miR-10b-5p	50 HC 31 CLD 27 HCC	blood	China	1.onco-miR2.liver injury	up	HCC from HC 0.85 HCC from CLD 0.73 CLD from HC 0.66	25761179^10^	NA
miR-122	miR-122-5p	89 HC 48 CHB 101 HCC	blood	China	liver injury	up	HCC from HC 0.79 CHB from HC 0.93	21229610^92^	NA
miR-122	miR-122-5p	167 HC 169 CHB 141 LC 457 HCC	blood	China	1.tumor size2.differentiation grade3.poor prognosis4.distance metastasis	down	0.864(training set) 0.888(validation set)	22105822^59^	NA
miR-122	miR-122-5p	15 HC 30 DN 120 HCC	serum	China	1.induce apoptosis2.suppress proliferation	up	0.629	26264553^44^	NA
miR-122	miR-122-5p	34 HC 70 HBV-HCC 48 CHB	serum	China	liver injury	up	HCC from HC 0.869 HBV-HCC from CHB 0.630	22174818^93^	NA
miR-122-5p	miR-122-5p	173 HC 233 LC 261 HCC	serum	China	1.regulating hepatocyte development and differentiation2.apoptosis and suppress proliferation	down	0.887(training sets) 0.879(validation sets)	25238238^86^	HepG2 and Hep3B cells
miR-1228-5p	miR-1228-5p	173 HC 233 LC 261 HCC	serum	China	NA	up	0.887(training sets) 0.879(validation sets)	25238238^86^	NA
miR-122a	miR-122-5p	85 volunteers matched	serum	China	tumor suppressor	down	0.707(0.943)^2^	23723713^98^	NA
miR-125b-5p	miR-125b-5p	28 HC 24 CHB 22 HBV-LC 20 HBV-HCC	plasma	Turkey	suppress the cell growth	up	NA	24595450^33^	AKT
miR-130a	miR-130a-3p	42 HC 125 HCV-CLD 112 HCV-HCC	blood	Egypt	NA	up	HCV-HCC from HC 0.91	26352740^42^	NA
miR-130b	miR-130b-3p	97 HCC	serum	China	onco-miR	up	0.914	22403344^134^	RUNX3
miR-139	miR-139-5p	31 CHB 31 HCC	plasma	China	1.suppress metastasis and progression of cancer cells2.tumor suppressor	down	HCC from CH 0.761 (0.770)^3^	24549282^51^	Rho-kinase 2
miR-141-3p	miR-141-3p	173 HC 233 LC 261 HCC	serum	China	NA	up	0.887(training sets) 0.879(validation sets)	25238238^86^	NA
miR-143	miR-143-3p	127 HC 118 CH 95 HCC	serum	China	differentiation	up	CH from HC 0.617 HCC from CH 0.795	24993656^62^	FNDC3B
miR-146a	miR-146a-5p	42 HC 125 HCV-CLD 112 HCV-HCC	blood	Egypt	1.suppresses HCC invasion2.exerted negative effects on anti-tumor immune response	up	HCV-HCC from HC 0.787 HCV-HCC from HCV-CLD 0.85	26352740^142^	VEGF
miR-146a	miR-146a-5p	313 HC 294 HCC	serum	China	onco-miR	NA	NA	24816919^107^	NA
miR-150	miR-150-5p	120 HC 110 CHB 120 HCC	serum	China	1.tumor suppressor2.metastasis3.BCLC stage4.advanced TNM stages	down	0.931	26215970^60^	NA
miR-150	miR-150-5p	15 HC 15 ICC	plasma	China	tumor suppressor	up	0.764	25482320^97^	NA
miR-15b	miR-15b-5p	96 HCC	serum	China	preventing replicative stress in response to mitogenicsignalling	up	0.98^4^	22403344^34^	NA
miR-16	miR-16-5p	107 CLD 105 HCC	serum	America	1.tumor suppressor2.apoptosis	down	NA	21278583^41^	BCL2, MCL1, CCND1, WNT3A
miR-17-5p	miR-17-5p	28 HC 26 CHC 30 HCV-positive cirrhosis 8 HCC	blood	Turkey	NA	up	NA	25391771^81^	NA
miR-181a	miR-181a-5p	50 HC 31 CLD 27 HCC	blood	China	tumor suppressor	down	HCC from HC 0.82 HCC from CLD 0.71 CLD from HC 0.64	25761179^10^	NA
miR-182	miR-182-5p	40 HC 95 BLD 103 HCC	serum	China	1.metastasis	up	0.911	25903466^61^	TP53INP1
miR-18a	miR-18a-5p	60 HC 30 HBV-CH 101 HBV-HCC	serum	China	1.liver injury2.onco-miR	up	NA	22865399^94^	NA
miR-192	miR-192-5p	167 HC 169 CHB 141 LC 457 HCC	blood	China	NA	up	0.864(training set) 0.888(validation set)	22105822^59^	NA
miR-192	miR-192-5p	42 HC 125 HCV-CLD 112 HCV-HCC	blood	Egypt	liver injury	up	HCV-HCC from HC 0.878 HCV-HCC from HCV-CLD 0.69	26352740^42^	NA
miR-192-5p	miR-192-5p	173 HC 233 LC 261 HCC	serum	China	NA	down	0.887(training sets) 0.879(validation sets)	25238238^86^	NA
miR-195	miR-195-5p	42 HC 125 HCV-CLD 112 HCV-HCC	blood	Egypt	1.onco-miR2.evading apoptosis3.tissue invasion and metastasis	down	HCV-HCC from HC 0.653 HCV-HCC from HCV-CLD 0.78	26352740^42^	FGF7 and GHR
miR-196a	miR-196a-5p	313 HC 294 HCC	serum	China	onco-miR	NA	NA	24816919^107^	NA
miR-199a-5p	miR-199a-5p	173 HC 233 LC 261 HCC	serum	China	tumor suppressor	down	0.887(training sets) 0.879(validation sets)	25238238^86^	NA
miR-19a	miR-19a-3p	42 HC 125 HCV-CLD 112 HCV-HCC	blood	Egypt	1.PV thrombosis2.invasion, satellite nodules and progression3.recurrence	down	HCV-HCC from HC 0.714 HCV-HCC from HCV-CLD 0.86	26352740^42^	NA
miR-206	miR-206	173 HC 233 LC 261 HCC	serum	China	NA	up	0.887(training sets) 0.879(validation sets)	25238238^86^	NA
miR-21	miR-21-5p	89 HC 48 CHB 101 HCC	blood	China	liver injury	up	HCC from HC 0.87 CHB from HC 0.91	21229610^92^	NA
miR-21	miR-21-5p	167 HC 169 CHB 141 LC 457 HCC	blood	China	tumor suppressor	up	0.864(training set) 0.888(validation set)	22105822^59^	PTEN
miR-21	miR-21-5p	50 HC 30 LC 136 HCC	serum	Japan	excessive secretion by primary cancer cells	up	CH from HC 0.773 HCC from HC 0.953	21749846^110^	NA
miR-21	miR-21-5p	30 HC 97 HCC	blood	China	1.promote cell proliferation2.tumor invasion	up	NA	25973032^55^	PDCD4 and PTEN
miR-21	miR-21-5p	74 ICC	serum	China	intrahepatic cholangiocarcinoma proliferation and growth	up	NA	25803229^56^	PTPN14 and PTEN
miR-215	miR-215	127 HC 118 CH 95 HCC	serum	China	metastasis	up	CH from HC 0.802 HCC from HC 0.816	24993656^62^	NA
miR-221	miR-221-3p	10 HC 30 HCV 30 HCV-LC 30 HCV-HCC	serum	Egypt	anti-apoptotic	down	0.655	25429320^43^	NA
miR-223	miR-223-3p	89 HC 48 CHB 101 HCC	blood	China	liver injury	up	HCC from HC 0.86 CHB from HC 0.88	21229610^92^	NA
miR-223-3p	miR-223-3p	28 HC 26 CHC 30 HCV-LC 8 HCC	blood	Turkey	NA	down	NA	25391771^81^	NA
miR-223-3p	miR-223-3p	28 HC 24 CHB 22 HBV-LC 20 HBV-HCC	plasma	Turkey	NA	down	NA	24595450^33^	NA
miR-24-3p	miR-24-3p	46 HC 31 CLD 84 HCC	serum	China	1.vascular invasion	up	HCC from CLD 0.636 (0.834)^5^	25129312^63^	NA
miR-26a	miR-26a-5p	167 HC 169 CHB 141 LC 457 HCC	blood	China	lower miR-26a expression experienced worse survival but better response to interferon therapy	down	0.864(training set) 0.888(validation set)	22105822^59^	NA
miR-26a-5p	miR-26a-5p	173 HC 233 LC 261 HCC	serum	China	NA	down	0.887(training sets) 0.879(validation sets)	25238238^86^	NA
miR-27a	miR-27a-3p	167 HC 169 CHB 141 LC 457 HCC	blood	China	onco-miR	down	0.864(training set) 0.888(validation set)	22105822^59^	NA
miR-296	miR-296-5p	42 HC 125 HCV-CLD 112 HCV-HCC	blood	Egypt	1.metastasis2.tumor angiogenesis	up	HCV-HCC from HC 0.792 HCV-HCC from HCV-CLD 0.645	26352740^42^	NA
miR-302c-3p	miR-302c-3p	28 HC 26 CHC 30 HCV-positive cirrhosis 8 HCC	blood	Turkey	NA	up	NA	25391771^81^	NA
miR-30c-5p	miR-30c-5p	28 HC 26 CHC 30 HCV-positive cirrhosis 8 HCC	blood	Turkey	1.HCV-positive cirrhosis2.interferon-beta therapy	up	NA	25391771^81^	NA
miR-331-3p	miR-331-3p	40 HC 95 BLD 103 HCC	serum	China	1.proliferation2.metastasis	up	0.89	25903466^61^	PH
miR-34a	miR-34a-5p	42 HC 125 HCV-CLD 112 HCV-HCC	blood	Egypt	child stage and BCLC score	up	HCV-HCC from HC 0.98 HCV-HCC from HCV-CLD 0.67	26352740^42^	NA
miR-375	miR-375	156 HC 78 HCC	serum	China	tumor suppressor	down	0.637	25618599^58^	NA
miR-375	miR-375	210 HC 135 HBV 48 HCV 120 HCC	serum	China	NA	up	0.96	21098710^140^	NA
miR-433-3p	miR-433-3p	173 HC 233 LC 261 HCC	serum	China	NA	up	0.887(training sets) 0.879(validation sets)	25238238^86^	NA
miR-483-5p	miR-483-5p	69 HC 69 HCC	serum	America	anti-apoptotic oncogene	up	HCC from HC 0.827	24127413^40^	NA
miR-885-5p	miR-885-5p	24 HC 23 CHB 26 LC 17 GC 9 ICC 6 FNH 46 HCC	serum	China	cholesterol reverse transport	up	0.904	20815808^111^	NA
let-7b	let-7b-5p	15 HC 30 DN 120 HCC	serum	China	tumor suppressor	up	0.645	26264553^44^	NA
miR-203	miR-203a-3p	10 HC 30 non-cirrhotic HCV 25 HCV-related cirrhosis 23 HCV-HCC	serum	Egypt	1.tumor-suppressive2.angiogenesis	down	HCC from non-HCC 0.76	27268654^141^	NA
miR-885-5p	miR-885-5p	192 HCC 96 LC 96 CHC 95 HC	serum	Egypt	1.onco-miR2.liver injury	up	HCC from HC 0.63 HCC from LC 0.775	27271989^120^	ISRE
miR-122	miR-122-5p	193 HCC 96 LC 96 CHC 95 HC	serum	Egypt	1.tumor suppressor2.regulate lipid and cholesterol metabolism	up	HCC from HC 0.617 HCC from LC 0.617	27271989^120^	ADAM17
miR-29b	miR-29b-3p	194 HCC 96 LC 96 CHC 95 HC	serum	Egypt	tumor suppressor	down	HCC from HC 0.766	27271989^120^	NA
miR-221	miR-221-3p	195 HCC 96 LC 96 CHC 95 HC	serum	Egypt	1.onco-miR2.apoptosis	up	HCC from LC 0.702	27271989^120^	CDKN1B/p27CDKN1C/p57
miR-181b	miR-181b-5p	196 HCC 96 LC 96 CHC 95 HC	serum	Egypt	1.onco-miR2.migration and invasion	up	HCC from LC 0.679	27271989^120^	TIMP3
miR-22	miR-22-3p	197 HCC 96 LC 96 CHC 95 HC	serum	Egypt	tumor suppressor	down	HCC from CHC 0.586	27271989^120^	HDAC4
miR-199a-3p	miR-199a-3p	198 HCC 96 LC 96 CHC 95 HC	serum	Egypt	tumor suppressor	down	HCC from CHC 0.7	27271989^120^	mTOR
miR-125b	miR-125b-5p	56 HC 63 CHB 59 HBV-LC 64 HBV-HCC	plasma	China	1.tumor suppressor2.migration and invasion3.cellular proliferation and cell cycle progression	down	HBV-HCC from HC 0.891	27152955^121^	LIN28B
miR-96	miR-96-5p	104 HCC 100 CHB 90 LC 120 HC	serum	China	1.onco-miR2.migration and invasion	up	HCC from CHB 0.803	26770453^142^	NA
miR-126	miR-126-3p	28 HC 20 LC 59 HCC	plasma	India	NA	up	low AFP HCC from non-HCC 0.765 low AFP HCC from LC 0.643	26756996^143^	APAF1, APC2, VEGFA, IRS1, CDKN2A
miR-224	miR-224-5p	26 HCC 22 LC 23 CHB 22 HC	serum	China	1.migration and invasion2.suppress apoptosis	up	0.88	26724963^144^	NA

Abbreviations and note: HC: healthy controls; CHB: patients with chronic type B hepatitis; CLD: chronic liver disease; HCV-CLD: non-malignant HCV-associated CLD patients; DN: chronic hepatitis B patients with pathologically proven DN; ICC: intrahepatic cholangiocellular carcinoma; LC: liver cirrhosis; HCV: hepatitis C virus HBV: hepatitis B virus; NA: not available; 1: upregulated in the HBV-LC group; 2: combined classifier (AFP and miRNA-122a); 3; combination of plasma miRNA-139 with serum AFP; 4: combined miR-15b and miR-130b; 5: Combined serum alpha-fetoprotein (AFP) and miR-24-3p.

**Table 3:** Prognostic biomarkers in tissues for hepatocellular carcinoma.

Reported ID	Offical ID	Sample	Ethnicity	Features	Expression	PMID	Validated Targets
miR-101	miR-101-3p	20 HC 25 HBV-HCC	China	1.HBsAg, HBV DNA level and tumor size	up	24260081^112^	NA
miR-101	miR-101-3p	130 HCC	China	tumor suppressor	down	23178713^99^	SOX9
miR-101	miR-101-3p	30 HC 79 CHB 61 HBV-LC 67 HBV-HCC	China	1.inhibit HCC cell proliferation2.tumor suppressor	up	24971953^38^	NA
miR-106b	miR-106b-5p	104 HCC	China	1.tumor size2. vascular invasion3. proliferation4. anchorage-independent growth of HCC cells5.metastasis	up	25466449^64^	NA
miR-122	miR-122-5p	60 HCC	China	1.tumor suppressor2.maintenance of normal physiological metabolism	down	26252254^100^	PKM2
miR-125b	miR-125b-5p	49 HCC	China	tumor suppressor	down	24811246^101^	Eif5a2
miR-1269	miR-1269a	95 HCC	China	1.tumor nodes2.portal vein tumor embolus3.vaso-invasion4.tumor capsular infiltration5.expression of MTDH6.onco-miR7.carcinogenesis, metastasis and invasion of HCC	up	25785048^72^	AGAP1, AGK, BPTF, C16orf74, DACT1, LIX1L, RBMS3, ZNF706 and BMPER
miR-128-3p	miR-128-3p	72 HCC	China	1.suppress proliferation2.suppress metastasis	down	25962360^73^	PIK3R1 PI3K/AKT
miR-130a	miR-130a-3p	102 HCC	China	1.gender, HBsAgstatus, tumor size, and TNM stage2.tumor suppressor	down	25218269^102^	NA
miR-137	miR-137	136 HCC	China	1.vein invasion2.distant metastasis3.inhibition promotes HCC cell growth	down	24970808^35^	AKT2
miR-146a	miR-146a-5p	85 HCC	China	tumor suppressor	down	24172202^103^	ROCK1
miR-155	miR-155-5p	100 HCC	China	1.metastasis2.inhibits apoptosis	up	23863669^45^	NA
miR-155	miR-155-5p	216 HCC	China	onco-miR	up	22629365^108^	NA
miR-17-5p	miR-17-5p	120 HCC	China	regulating proliferation and migration	up	22583011^65^	p38 MAPK-HSP27
miR-182	miR-182-5p	81 HCC	China	1.onco-miR2.motility and invasiveness	up	25813403^66^	FOXO1
miR-182	miR-182-5p	86 HCC	China	intrahepatic metastasis	up	22681717^67^	MTSS1
miR-183	miR-183-5p	81 HCC	China	1.onco-miR2.motility and invasiveness	up	25813403^66^	FOXO1
miR-185	miR-185-5p	41 NTR 54 TR	China	1.suppress the tumor cell growth2.suppress invasive	down	23648054^36^	NA
miR-188-5p	miR-188-5p	250 HCC	China	1.suppress tumor cell proliferation2.suppress metastasis	down	25998163^74^	FGF5
miR-18b	miR-18b-5p	110 HCC	Japan	1.proliferation2.loss of cell adhesion ability	up	23496901^52^	TNRC6B
miR-199a-5p	miR-199a-5p	120 HCC	China	1.Negatively Associated With Malignancies2.Regulates Glycolysis3.Lactate Production	down	26054020^145^	Hexokinase 2
miR-206	miR-206	147 HCC	China	1.suppresses cell proliferation2.promotes apoptosis.	down	25513086^46^	NA
miR-21	miR-21-5p	50 HC 30 CH 136 HCC	Japan	NA	down	21749846^110^	NA
miR-21	miR-21-5p	112 HCC	China	1.tumor differentiation2.TNM stage3.vein invasion	up	26261620^68^	NA
miR-21	miR-21-5p	119 HCC	China	1.tumorinvasion, metastasis and prognosis2.promote cell proliferation and invasion3.inhibits cell apoptosis	up	25150373^47^	NA
miR-21	miR-21-5p	74 ICC	China	intrahepatic cholangiocarcinoma proliferation and growth	up	25803229^56^	PTPN14 and PTEN
miR-212	miR-212-3p	86 HCC	China	1.inhibited cell proliferation2.induced apoptosis	down	26347321^48^	FOXA1
miR-214	miR-214-3p	65 HCC	China	tumor suppressor	down	23962428^104^	FGFR-1
miR-25	miR-25-3p	96 HCC	Iran	1.TNM stage2.suppress proliferation3.suppress migration	up	26209296^69^	NA
miR-26a	miR-26a-5p	120 HCC	China	1.Cell Cycle2.angiogenesis	up	24259426^84^	CDK6, cyclin D1
miR-26a	miR-26a-5p	130 HCC	China	1.suppress the tumor cell growth2.suppress invasive	down	23389848^37^	interleukin-6-Stat3
miR-331-3p	miR-331-3p	457 HCC	China	1.Promotes Proliferation2.Metastasis	up	24825302^70^	Leucine-Rich Repeat Protein Phosphatase
miR-34a	miR-34a-5p	120 HCC	China	1.tumor size2.higher serum AFP level	down	25596083^113^	NA
miR-424	miR-424-5p	96 HCC	China	suppressed proliferation	down	26315541^87^	pRb-E2F pathway, Akt3 and E2F3
miR-503	miR-503-5p	20 HCC	China	suppress metastasis	down	26163260^75^	PRMT1
miR-744	miR-744-5p	96 HCC	China	1.tumour suppressor2.tumor malignancy3.tumor cell proliferation4.invasion and migration5.HCC recurrence6.poor prognosis	down	25543521^76^	NA
miR-9	miR-9-5p	200 HCC	China	1.tumour suppressor2.tumor stage3.venous infiltration	up	25552204^71^	NA
miR-96	miR-96-5p	81 HCC	China	1.onco-miR2.motility and invasiveness	up	25813403^66^	FOXO1
miR-125a	miR-125a-5p	80 HCC	China	1.Proliferation 2.Metastasis	down	22768249^146^	MMP11 and VEGF
miR-99a	miR-99a-5p	142 HCC	China	tumor suppressor	down	21878637^147^	NA

Abbreviations and note: HC: healthy controls; CHB: patients with chronic type B hepatitis; CLD: chronic liver disease; HCV-CLD: non-malignant HCV-associated CLD patients; DN: chronic hepatitis B patients with pathologically proven DN; ICC: intrahepatic cholangiocellular carcinoma; LC: liver cirrhosis; HCV: hepatitis C virus; HBV: hepatitis B virus; CH: chronic hepatitis; TR: treated recurrence group; NTR: none treated recurrence group; NA: not available.

**Table 4:** Prognostic biomarkers in blood for hepatocellular carcinoma.

Reported ID	Offical ID	Sample	Source	Ethnicity	Features	Expression	PMID	Validated Targets
miR-1	miR-1-3p	54 LC 195 HCC	serum	Germany	1.differentiation2.tumor suppressor	up	23810247^91^	NA
miR-101	miR-101-3p	20 HC 25 HBV-HCC	serum	China	1.HBsAg, HBV DNA level and tumor size	up	24260081^112^	NA
miR-101	miR-101-3p	30 HC 79 CHB 61 HBV-LC 67 HBV-HCC	serum	China	1.inhibit HCC cell proliferation2.tumor suppressor	up	24971953^38^	NA
miR-122	miR-122-5p	122 HCC	blood	China	1.tumor suppressor2.proliferation3.differentiation4.regulation of cholesterol and lipid metabolisms5.stability and propagation of hepatitis C virus and hepatitis B infection	up	25636448^77^	NA
miR-122	miR-122-5p	120 HCC	plasma	South Korea	1.hepatic necroinflammatory activity2.cell death3.tumor suppressor	up	26129878^49^	NA
miR-122	miR-122-5p	54 LC 195 HCC	serum	Germany	1.liver transaminases2.MELD score	down	23810247^91^	NA
miR-128-2	miR-128-2	20 HCC 20 HCC(PVTT)	serum	China	onco-miR	up	25642945^109^	NA
miR-150	miR-150-5p	120 HC 110 CHB 120 HCC	serum	China	1.tumor suppressor2. metastasis3.BCLC stage4.advanced TNM stages	down	26215970^60^	NA
miR-16	miR-16-5p	60 HC 90 HCC	serum	China	1.tumor size2.liver dysfunction and coagulation defect	down	24697119^114^	NA
miR-16	miR-16-5p	40 HCV 40 HCC	serum	Egypt	1.apoptosis2.bilirubin	down	26133725^50^	NA
miR-17-5p	miR-17-5p	96 HCC	blood	China	1.metastasis2.TNM stage	up	23108086^78^	NA
miR-182	miR-182-5p	40 HC 95 BLD 103 HCC	serum	China	metastasis	up	25903466^61^	TP53INP1
miR-199a	miR-199a-5p	40 HCV 40 HCC	serum	Egypt	tumor size	down	26133725^50^	NA
miR-203a	miR-203a-3p	90 HCV 152 HCV-HCC	serum	China	tumor suppressor	down	26210453^105^	Snal2
miR-21	miR-21-5p	50 HC 30 CH 136 HCC	serum	Japan	NA	down	21749846^110^	NA
miR-21	miR-21-5p	74 ICC	serum	China	intrahepatic cholangiocarcinoma proliferation and growth	up	25803229^56^	PTPN14 and PTEN
miR-21	miR-21-5p	60 HC 90 HCC	serum	China	liver injury	down	24697119^114^	NA
miR-24-3p	miR-24-3p	46 HC 31 CLD 84 HCC	serum	China	vascular invasion	up	25129312^63^	NA
miR-30c	miR-30c-5p	90 HCV 152 HCV-HCC	serum	China	tumor suppressor	down	26210453^105^	EMT
miR-331-3p	miR-331-3p	40 HC 95 BLD 103 HCC	serum	China	1.proliferation2.metastasis	up	25903466^61^	PH
miR-335	miR-335-5p	125 HC 125 HCV/HBV 125 HCC	serum	China	response to TACE and clinical outcome	down	26305026^148^	NA
let-7f	let-7f-5p	60 HC 90 HCC	serum	China	1.tumor size2.early recurrence	down	24697119^114^	NA

Abbreviations and note: PVTT: portal vein tumor thrombosis; LC: liver cirrhosis; HBV: Hepatitis B Virus; HCV: Hepatitis C Virus; HC: healthy controls; CHB: patients with chronic type B hepatitis; BLD: benign liver diseases; ICC: intrahepatic cholangiocellular carcinoma; CH: chronic hepatitis; NA: not available.

**Table 5:** Therapeutic biomarkers for hepatocellular carcinoma.

Reported ID	Offical ID	Sample	Source	Ethnicity	Features	Expression	PMID	Validated Targets
miR-335	miR-335-5p	62 HCC	tissue	China	inhibit the proliferation and migration invasion	down	25804796^149^	ROCK1
miR-192	miR-192-5p	59 HC 59 HCC	tissue	South Korea	increase tumor cell migration and invasion	down	25065598^150^	NA
miR-224	miR-224-5p	9 HC 10 HCC	tissue	China	1.cell proliferation s2. migration3.invasion4.anti-apoptosi	up	24789420^39^	CD40
miR-214	miR-214-3p	9 HC 10 HCC	tissue	China	tumor suppressor	down	24789420^39^	EZH2, CTNNB1 and CDH1
miR-148a	miR-148a-3p	19 HCC	tissue	China	onco-miR	up	22496917^106^	NA
miR-206	miR-206	147 HCC	tissue	China	1. suppress cell proliferation2.promote apoptosis.	down	25513086^46^	NA
miR-331-3p	miR-331-3p	457 HCC	tissue	China	1. promote proliferation2. metastasis	up	24825302^70^	Leucine-Rich Repeat Protein Phosphatase
miR-26a	miR-26a-5p	120 HCC	tissue	China	1. cell Cycle2. angiogenesis	up	24259426^84^	CDK6, cyclin D1
miR-26a	miR-26a-5p	130 HCC	tissue	China	1. suppress the tumor cell growth2. suppress invasive	down	23389848^37^	interleukin-6-Stat3

Abbreviations and note: HC: healthy controls; NA: not available.
